# An integrative systems biology strategy to support the development of adverse outcome pathways (AOPs): a case study on radiation-induced microcephaly

**DOI:** 10.3389/fcell.2023.1197204

**Published:** 2023-06-22

**Authors:** Thomas Jaylet, Roel Quintens, Olivier Armant, Karine Audouze

**Affiliations:** ^1^ Université Paris Cité, Inserm T3S, Paris, France; ^2^ Belgian Nuclear Research Centre, SCK CEN, Mol, Belgium; ^3^ PSE-ENV/SRTE/LECO, Institut de Radioprotection et de Sûreté Nucléaire (IRSN), Saint-Paul-Lez-Durance, France

**Keywords:** adverse outcome pathways, AOP, AOP-helpFinder, text mining, integrative systems biology, biological networks, microcephaly, ionizing radiation

## Abstract

Adverse Outcome Pathways (AOPs) are useful tools for assessing the potential risks associated with exposure to various stressors, including chemicals and environmental contaminants. They provide a framework for understanding the causal relationships between different biological events that can lead to adverse outcomes (AO). However, developing an AOP is a challenging task, particularly in identifying the molecular initiating events (MIEs) and key events (KEs) that constitute it. Here, we propose a systems biology strategy that can assist in the development of AOPs by screening publicly available databases, literature with the text mining tool AOP-helpFinder, and pathway/network analyses. This approach is straightforward to use, requiring only the name of the stressor and adverse outcome to be studied. From this, it quickly identifies potential KEs and literature providing mechanistic information on the links between the KEs. The proposed approach was applied to the recently developed AOP 441 on radiation-induced microcephaly, resulting in the confirmation of the KEs that were already present and identification of new relevant KEs, thereby validating the strategy. In conclusion, our systems biology approach represents a valuable tool to simplify the development and enrichment of Adverse Outcome Pathways (AOPs), thus supporting alternative methods in toxicology.

## 1 Introduction

The Adverse Outcome Pathway (AOP) is a conceptual framework developed to address some of the toxicological and ecotoxicological challenges of the 21st century (Ankley et al, 2010). AOPs allow to organize toxicological and ecotoxicological data as a linear combination of biological events triggered by exposure to a stressor (e.g., pollutants, chemicals, physical stress). An AOP starts with a molecular initiating event (MIE), progresses via a cascade of causal key events (KEs) through different levels of biological organization (cells, tissues, organs, individual) to the appearance of an Adverse Outcome (AO). Biological events (MIE, KE, AO) are not exclusive to one AOP and can be shared by a multitude of AOPs thereby allowing the assembly of complex Adverse Outcome Networks (AONs). These concepts allow to better evaluate and in combination with Aggregate Exposure Pathway (Teeguarden et al, 2016), identify the risks caused by exposure to stressors and mixtures of stressors on human health and on the environment and help reduce the use of animal testing methods (Burden et al, 2015).

Given the enormous amount of existing knowledge in the scientific literature and other data sources, the identification of relevant biological information to build an AOP is a complex and time-consuming task. In order to facilitate this step and save time, the tool AOP-helpFinder, based on artificial intelligence was recently developed (Carvaillo et al, 2019; Jornod et al, 2022). AOP-helpFinder uses text mining and graph theory to automatically explore the literature available in the PubMed database to identify known linkages between stressors and KEs. This tool was successfully used to gather knowledge for several AOP including the activation of AhR leading to breast cancer (AOP 439, https://aopwiki.org/aops/439) (Benoit et al, 2022) and AOP on radiation-induced microcephaly (AOP 441, https://aopwiki.org/aops/441) (Jaylet et al, 2022). For the latter AOP, its development required the consultation of an expert group within the European project RadoNorm (https://www.radonorm.eu/) in order to identify relevant KEs and find consensus about the weight of evidence of their causal relationships (Jaylet et al, 2022).

Despite the utility of this method to search and compile existing biological knowledge, the *a priori* knowledge of the potential KEs that could constitute the AOPs is still required. In addition, this approach identifies and compiles information only from the literature. As it may be difficult to objectively predetermine all the KEs that can constitute an AOP through expert consensus, several complementary sources of information should be considered. In this context, we propose an innovative integrative systems biology strategy allowing the identification of potential KEs for the development or enrichment of AOPs. Systems biology allows to statistically integrate complex biological data from several sources in an automated manner to build computational models taking into consideration the different levels of biological organization. Various systems biology models were previously developed, such as predictive models for endocrine disruptors and their associations to COVID-19, for human systems affected by exposure to environmental chemicals or to assess the chemical etiologies of diabetes (Wu et al, 2021). In the context of AOP development, combining knowledge from various data sources (protein-protein interactions, pathway databases, CompTox, literature,…) allowed to build a comprehensive complex framework linking the stressor bisphenol F to thyroid malignancies (Rugard et al, 2020). Recently, a study applied radiation omics in the development of an AOP network for radiation-induced cardiovascular disease (Azimzadeh et al, 2022).

In this study, we performed an integrative systems biology strategy to investigate the previously established AOP on microcephaly triggered by prenatal exposure to ionizing radiation (AOP 441). This strategy is easy to apply as it only requires inputting the specific AO and the corresponding stressor(s) that trigger the AOP. By integrating text mining of scientific literature, biological enrichment analyses and network analysis considering integration and statistics on large amount of data from several databases (i.e., GeneCards, DisGeNET, PubMed), the use of this approach in the current study aims to identify the genes and pathways implicated in the process of radiation-induced microcephaly. Microcephaly is one of the most prevalent neurodevelopmental disorders characterized by reduced brain size and frequently accompanied by intellectual deficits (Becerra-Solano et al, 2021). Numerous cases of microcephaly have been observed following *in utero* exposure to radiation from nuclear bomb in Hiroshima and Nagasaki (Plummer, 1952; Miller and Blot, 1972; Otake and Schull, 1998). Additionally, abnormal rates of microcephaly continue to be observed in contaminated areas of Ukraine affected by the Chernobyl incidents and exhibiting high levels of Cesium-137 (Wertelecki et al, 2018). Cases of reduced brain size have also been documented on wildlife in birds living in the Chernobyl-affected zones and in Japanese monkeys fetus following the Fukushima disaster (Møller et al, 2011; Hayama et al, 2017). A Consensus exists that production of neurons from neuronal progenitors pool (i.e., neurogenesis) is altered after exposure to ionizing radiation. Therefore, understanding all the mechanisms involved in neurogenesis disruption following exposure to ionizing radiation is of notable importance for risks estimation of the stress response during the critical and sensitive stage of embryonic development. Thus, applying our computational approach to this case study serves as a relevant example to validate the strategy and allows for the rapid identification of novel KEs complementing those already identified by experts.

## 2 Materials and methods

**FIGURE 1:**
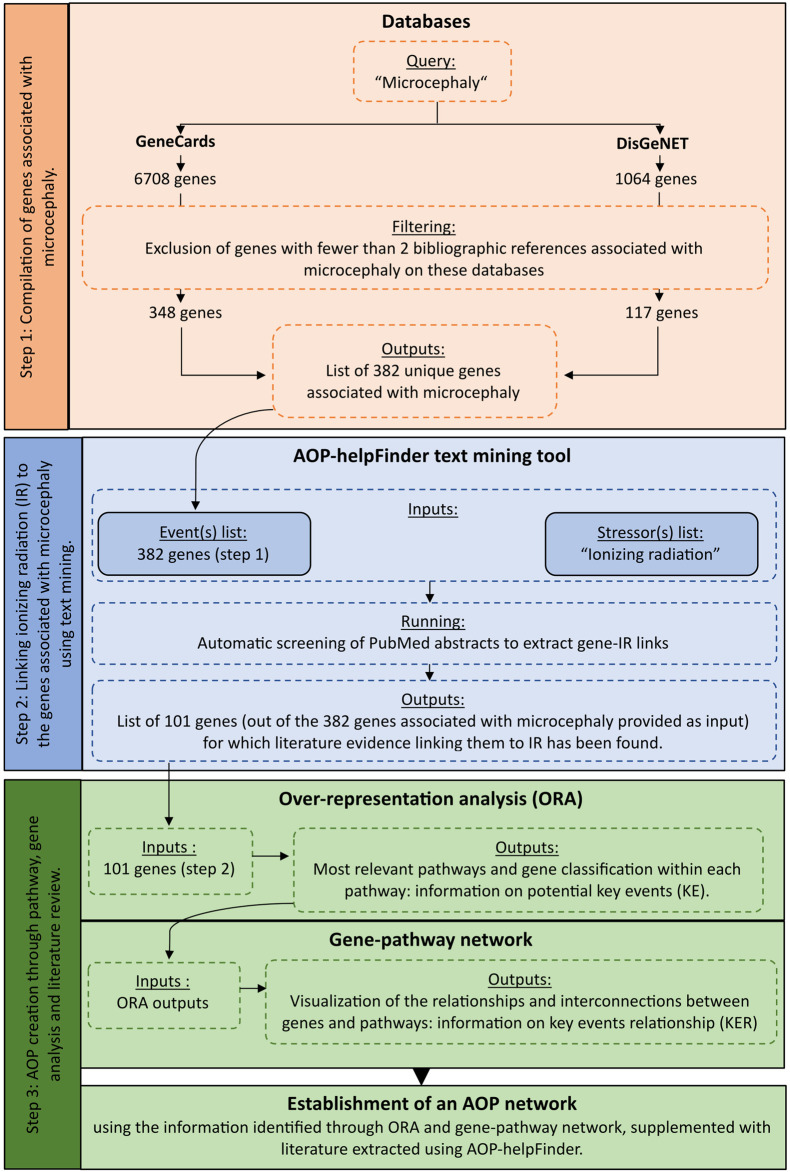
Detailed workflow on the systems biology procedure applied to radiation-induced microcephaly for AOP development.

To identify complementary biological events to improve AOP construction, we developed an integrative systems biology strategy divided into three steps (Figure 1).

### 2.1 Gene-microcephaly associations

The first step of this approach consisted in identifying and extracting a list of genes known to be associated with the Adverse Outcome (AO) of interest, which in our case was microcephaly, by querying various public databases. The selected databases for this analysis were GeneCards (https://www.genecards.org/- version 5.11) and DisGeNET (https://www.disgenet.org/- version 7.0). GeneCards is an integrative database that provides information on all annotated and predicted human genes by aggregating different web sources. DisGeNET is a database that integrates a collection of genes associated with human diseases and compiles more than 20,000 unique human genes and orthologs from various sources, including human/animal databases and text mining. In both databases, the results are sorted based on a score (gene disease association—gda score for DisGeNET and Relevance score for GeneCards) that takes into account various information such as curation level, organism, the different resources and the number of publications supporting the association between the gene and the disease.

These two databases were queried using the term “Microcephaly” (corresponding to our AO of interest), and all genes associated with this query were downloaded. Since the process of neurogenesis is well conserved across vertebrate species, we decided to integrate orthologs (rat/mouse) to retrieve as much important information as possible. To facilitate data integration and avoid potential redundancy between species, all genes were mapped to their HUGO symbols using the HGNC Comparison of Orthology Predictions (HCOP) tool (https://www.genenames.org/tools/hcop/).

To improve the confidence level of our study and retain only the most relevant genes for our analysis, we applied a filter based on the number of publications and excluded any genes with fewer than 2 scientific publications associated with microcephaly referenced on GeneCards and DisGeNET. The resulting gene list after the exclusion filter served as input for the next step of text mining (Figure 1; Supplementary Table S1).

### 2.2 Gene-stressor linkages

The second step aims to establish a connection between the genes extracted in the first step and potentially known stressors using contained in the abstracts of PubMed literature. In our case, we aimed to associate our list of genes associated with microcephaly with exposure to ionizing radiation (IR). To achieve this, we employed AOP-helpFinder (v1.0), a hybrid tool that combines text mining and graph theory. This tool can be accessed online at http://aop-helpfinder.u-paris-sciences.fr/index.php (accessed on 22 July 2022). This tool is specifically designed to identify and extract connections between co-occurring terms, including stressor-event or stressor-gene relationships (Gundacker et al, 2022). The program converts sentences into a graph structure, where words are depicted as nodes connected by weighted edges that reflect the proximity between words. This graph-based representation allows AOP-helpFinder to calculate scores, taking into account the distance between words (distance score) and their position in the abstract. As a result, it allows for capturing and retaining the most pertinent links.

The tool requires two inputs to operate.

1. A list of key events, which in this case corresponds to our list of genes associated with microcephaly extracted from the GeneCards and DisGeNET databases (step 1).
2. A list of stressors. Since AOP 441 is specifically related to “ionizing radiation” on AOP-Wiki, which includes various radionuclides (e.g., uranium, cesium, etc.) and types of radiation (e.g., low LET radiation, X-rays, gamma rays, etc.), we chose to use only this term as the input for the stressor list, comprising over 100,000 articles from PubMed.

Using these inputs, AOP-helpFinder automatically analyzes the entire PubMed abstracts related to IR to identify connections with the list of genes associated with microcephaly. To enhance confidence in the obtained results, we configured the “Reduced search” parameter to 20%. This option, based on the position of the links in the abstract, provides fewer results compared to the default parameter (0%), but it significantly reduces the rate of false positives by excluding links found in the first 20% of abstracts, which often correspond to the introduction or literature review sections rather than results.

As output, AOP-helpFinder provides a collection of articles that support the association between genes and IR. This text mining step plays a crucial role in our strategy as it enables the identification of biological knowledge that connects IR to microcephaly, thereby providing more detailed mechanistic information.

### 2.3 Pathway enrichment for microcephaly-linked data and network development

The last step was the enrichment and analyses of all the extracted data to identify potential KEs of the AOP, from the biological information linking IR and microcephaly. A biological enrichment analysis was performed with g:Profiler enrichment tool (Raudvere et al, 2019) (version *e107_e.g.,54_p17_bf42210)* using the list of selected genes common to IR and microcephaly in order to identify the most impacted pathways. In this approach, biological enrichment differs from its original use, as the list of genes extracted in the previous steps is provided as input, without considering any expression level. In systems biology, this strategy has already been applied to complement and classify the extracted data (Rugard et al, 2020; Wu et al, 2021). The over representation analysis (ORA) was done by querying different databases: KEGG (Kyoto Encyclopedia of Genes and Genomes—2022/09 release) and REACTOME (2022/09 release), which contain a collection of manually curated pathways as well as Wikipathways (2022/09 release), a community platform where pathways are curated (as of 2023, January). A hypergeometric test, corrected by the Benjamini–Hochberg method was done to adjust *p*-values. A significance level of 0.05 for the adjusted *p*-values was used to select significant pathways. In addition to the enrichment results, a gene-pathway network was created using KEGG and Reactome classification. The important identified pathways may thus correspond to Key Events leading to microcephaly, and the genes classified in these pathways may provide information on the Key Event Relationships (KERs). After identifying relevant information through the enrichment and the network analyses, a manual curation by experts of the full text articles identified by AOP-helpFinder was performed to provide the mechanistic and biological information leading to radiation-induced microcephaly (Figure 1).

## 3 Results

### 3.1 Identification of genes involved in the progression of microcephaly

The screening of both the GeneCards and the DisGeNET databases, allowed the identification of 6,708 and 1,064 genes respectively, described as being associated in the progression of microcephaly. As more than 80% of these genes have no bibliographic supports, we increased the level of confidence and the robustness of this dataset by keeping only the protein-coding genes with at least two scientific publications describing an association with microcephaly (Supplementary Figure S1). This led to the identification of 348 genes from GeneCards and 117 genes from DisGeNET, corresponding to a total of 382 unique genes linked to microcephaly progression (Supplementary Table S1). It is important to note that no causative relationship between the gene and microcephaly should be expected from this screening, although this method could identify the majority of currently known primary hereditary microcephaly (MCPH) genes (Siskos et al, 2021). For example, the genes MCPH1, ASPM (MCPH5), CENPJ (MCPH6), WDR62 (MCPH2), and CDK5RAP2 (MCPH3) are among the genes with the highest gda score (DisGeNET) and Relevance score (GeneCards), indicating their significant association with microcephaly as evidenced by a large number of publications referenced in both databases (Supplementary Table S1).

### 3.2 Identification of gene-IR links by text mining

**FIGURE 2:**
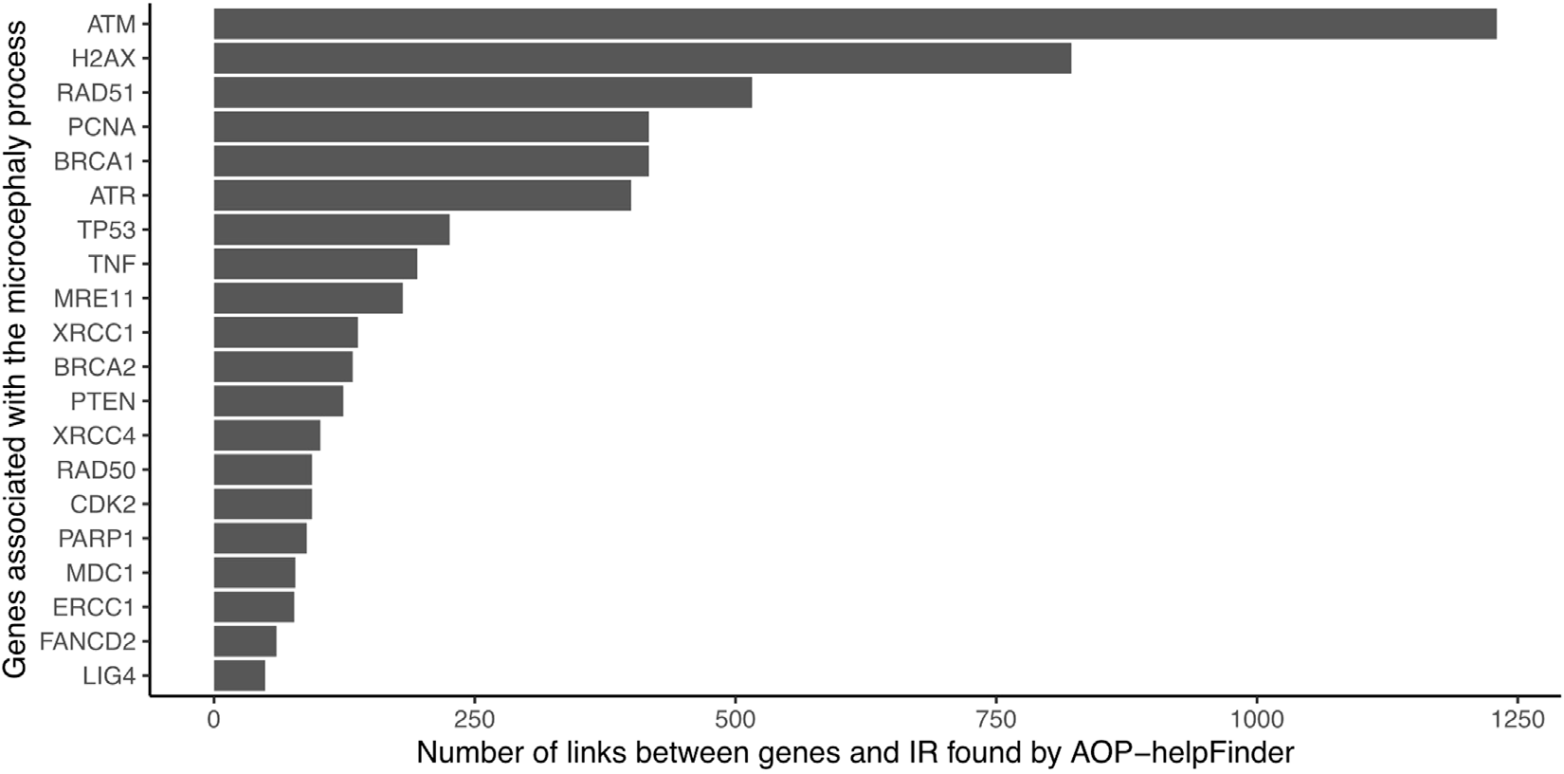
Number of gene-IR links identified by AOP-helpFinder (07/2022). Only the 20 genes with the most links are shown in the figure.

The 382 unique genes were combined with the stressor “Ionizing radiation” as inputs for the text mining tool AOP-helpFinder. Through a comprehensive analysis of 131,781 abstracts related to IR, which were extracted from a pool of 34 million abstracts available in the current PubMed database, AOP-helpFinder successfully established a link between 101 genes and IR (Supplementary Table S1). We found 20 genes with more than 50 links, including many known genes associated in the progression of radiation-induced microcephaly (Figure 2). For instance, the tumor suppressor gene TP53, a major key event in the microcephaly AOP, was linked to over 400 articles on IR. Additionally, several genes involved in the DNA damage response, another key event in AOP 441, were found to have multiple links to IR, including ATM (over 1,200 links), ATR, H2AX, and BRCA1 (Jaylet et al, 2022). These genes have also been well studied in the context of microcephaly, with 9 articles describing a link between TP53 and microcephaly, and 5 articles describing a link between ATM and microcephaly according to GeneCards (Supplementary Table S1), demonstrating consistency between the results of this case study and those verified in AOP 441.

### 3.3 Identification of common key events to the AOP 441 “radiation-induced microcephaly” by pathway analyses

The proposed *in-silico* strategy allowed the identification of 101 genes associated with biological processes that can lead to the progression of radiation induced microcephaly. We performed a biological enrichment analysis on this gene list, by querying different pathway databases (KEGG; Reactome, Wikipathways), to identify the most represented biological pathways associated with radiation-induced microcephaly, and thus to identify potential novel KEs.

**FIGURE 3:**
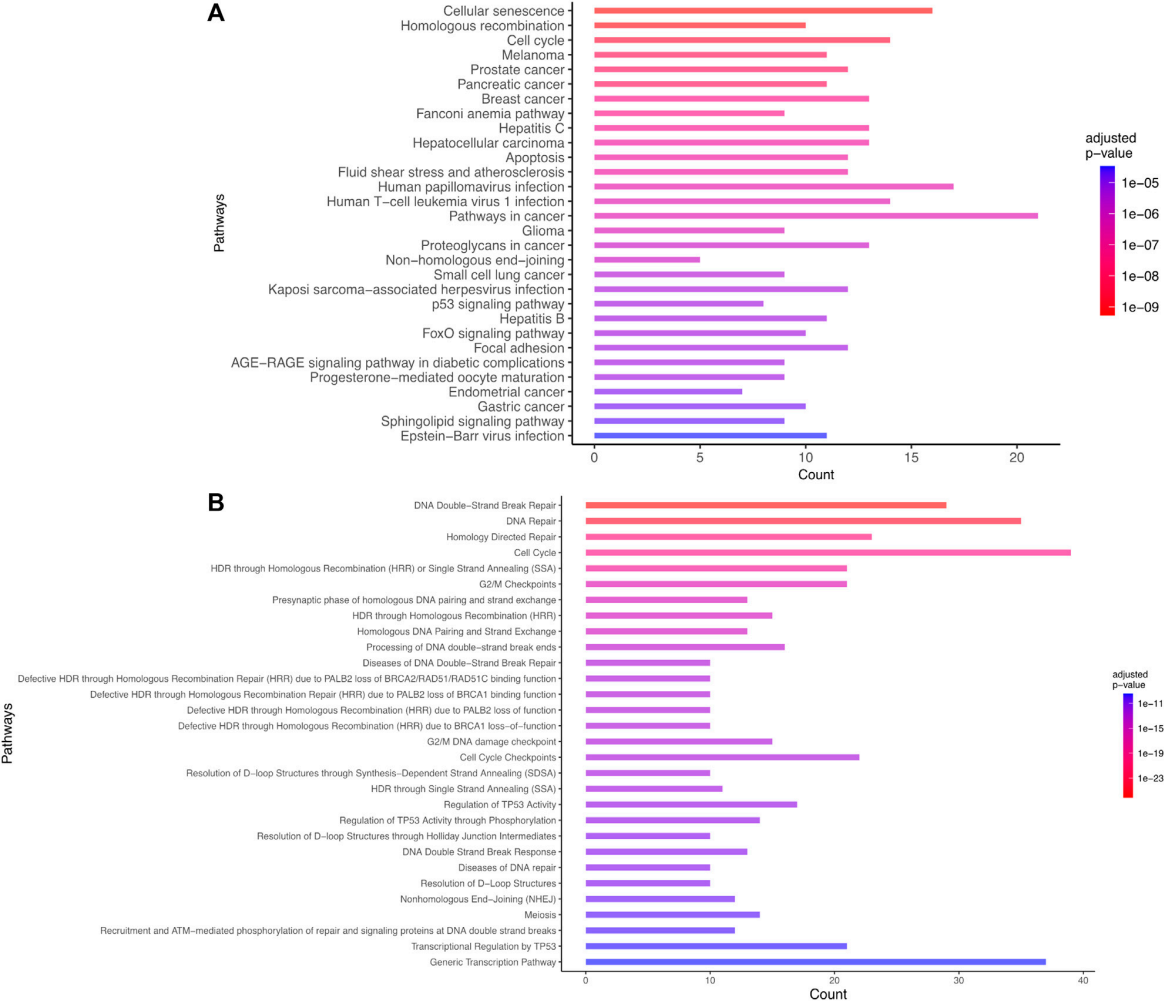
Biological enrichment results using KEGG (2022/09 release) **(A)** and Reactome (2022/09 release) **(B)** for the list of 101 genes common to microcephaly and ionizing radiation extracted by a computational strategy. Only the top 30 terms are shown.

The most statistically significant results corresponded to pathways known to be involved in the response to IR. Particularly, a set of pathways associated with DNA damage and repair (such as double strand breaks DSB repair, DNA repair, DSB response with corresponding adjusted *p*-values ranging between 8e-27 and 8e-12 on Reactome), pathways associated with P53 (e.g., Regulation of TP53 activity, P53 signaling pathway with adjusted *p*-values lower than 1e-11 on Reactome and 1e-5 on KEGG), apoptosis (adjusted *p*-value = 2e-7 for KEGG) were identified (Supplementary Table S2). The current preliminary AOP for microcephaly (AOP 441) is composed of the following KEs: “DNA damage” inducing “Activation of TP53” leading to “Apoptosis” and “Premature cell differentiation” leading to “Microcephaly.” These findings confirm the efficiency of the automated strategy to identify known KE relevant for AOP development (Figure 3; Supplementary Figure S2).

### 3.4 Development of a network-based model for microcephaly

We next constructed a network linking the different enriched pathways via the genes involved in these pathways. Indeed, genes can be part of different pathways. Thus, the created gene-pathway network allowed to explore the relationships between KEs and to identify genes potentially involved in KERs. For example, there is a large panel of genes involved in both the response to radiation-induced DNA damage and TP53 activation. Among the genes coding for proteins important for the DNA-damage response, the sensor genes MRE11 and Rad50 of the MRN complex were found, as well as important transducers such as ATM or ATR and ATRIP and mediators (MDC1, TP53BP1, BRCA1, RBBP8, Checkpoints Kinases and histone H2AX linked to DNA damage) (Figure 3).

Therefore, these results show that this automated strategy quickly identifies biological pathways known to be involved in radiation-induced microcephaly. In addition, it identifies genes and potential molecular mechanisms involved in the considered enriched biological process thereby providing a basis for the description of KERs. However, it is important to stress that this approach does not necessarily provide causal or mechanistic links between genes, pathways and disease. Rather, the use of AOP-helpFinder combined with these analyses can provide a rapid prioritization of the knowledge from the relevant literature to further explore potential mechanistic links leading to radiation-induced microcephaly. Based on the evidence automatically gathered here, the KER describing the relationship between the KEs “DNA damage” and “TP53 activation,” can be the activation via phosphorylation of ATM following sensing DNA double strand breaks (DSB) (Uziel et al, 2003). Indeed, the activation of ATM leads to the activation of various pathways related to TP53, including the histone H2AX, as well as various mediators such as MDC1 and TP53BP1 which will accumulate at DNA damage sites following ATM activation (Burma et al, 2001; Goldberg et al, 2003; Lou et al, 2006). All these genes, critical in the cellular response to DNA damage, were detected by AOP-helpFinder as part of the pathways induced by IR and possibly linked to radiation-induced microcephaly if damages are not efficiently repaired. This cascade of events finally leads to the activation of effector kinases (e.g., checkpoint kinases) and tumor suppressor protein TP53, which will have a key role in inducing cell cycle arrest and in the choice between DNA repair, apoptosis, senescence or differentiation depending on the degree of DNA damage (Banin et al, 1998; Turenne et al, 2001; Fei and El-Deiry, 2003).

### 3.5 Identification of new KEs and KERs: enrichment of the AOP 441

**FIGURE 4:**
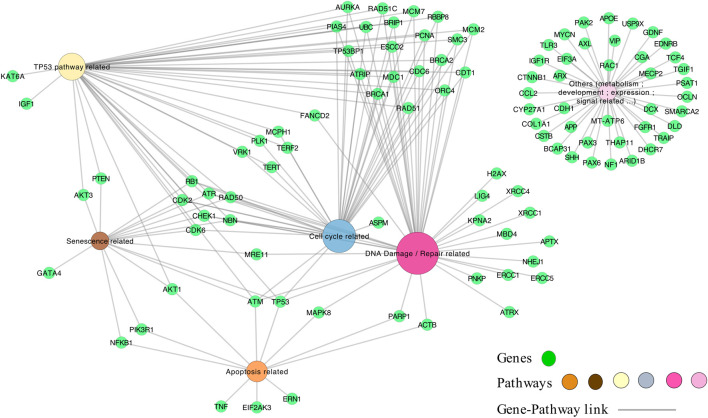
The genes-pathways network. The genes correspond to the 101 genes (green nodes) common to microcephaly and ionizing radiation extracted by the computational strategy, and are linked to the pathways (multi-colors) according to their classification in the pathways by KEGG and Reactome.

**FIGURE 5:**
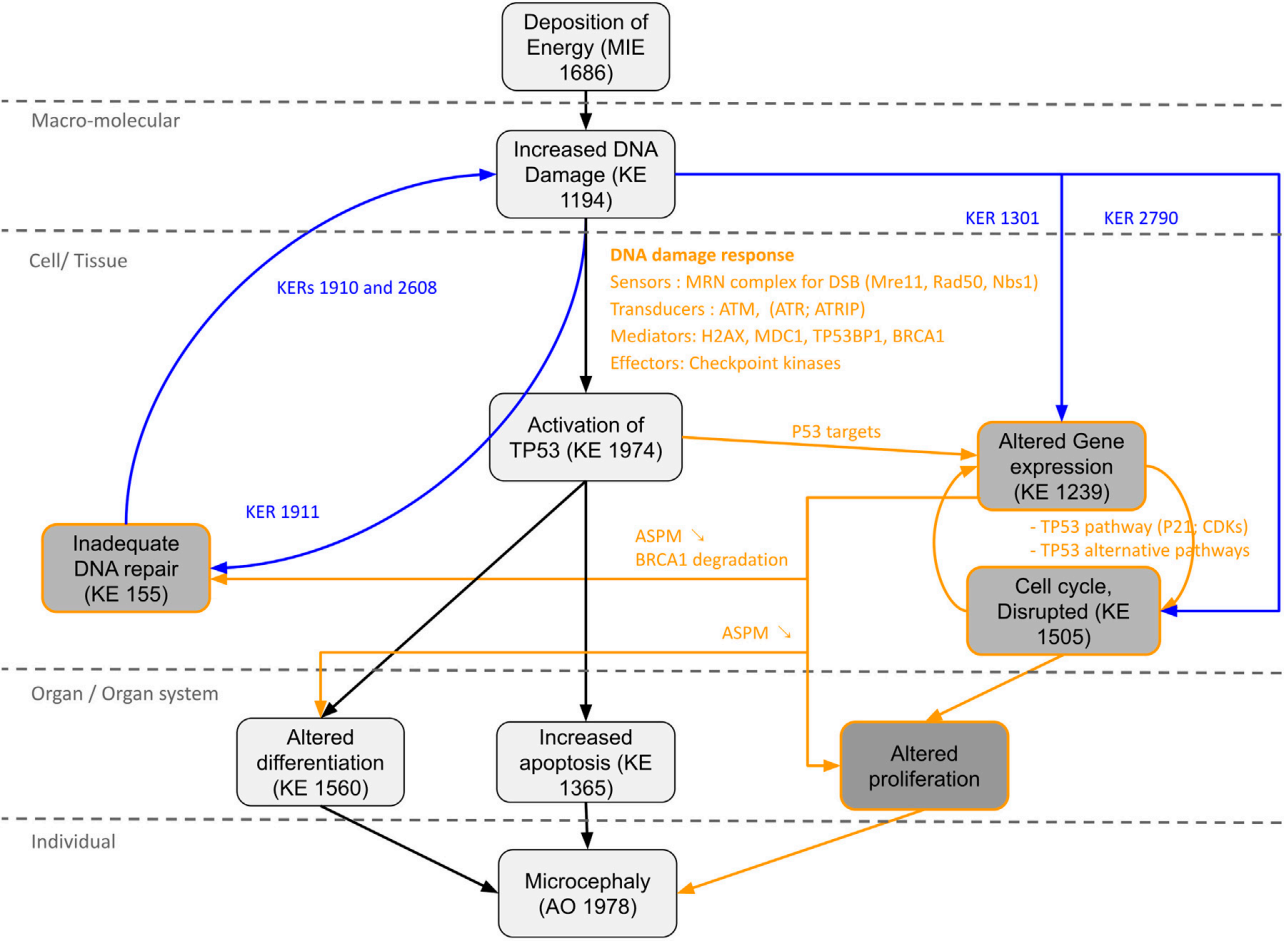
Proposal AOP network (AON) to complement the AOP 441“Ionizing radiation-induced DNA damage leads to microcephaly via apoptosis and premature cell differentiation.” The different Key Events (KEs) and Key Events Relationship (KERs) circled in black correspond to the information already present in the AOP. The KEs, KERs and orange writing correspond to the new information brought by the computational strategy. The KERs and blue writing correspond to the links already described on AOP-Wiki, which were used to enrich the network.

Novel potential KEs, not described in the preliminary AOP 441, were also identified by this non-supervised strategy. Among these, several genes involved in the cell cycle (adjusted *p*-value = 1.6e-9 for KEGG and 1.43e-19 Reactome) or senescence (adjusted *p*-value = 5.48e-10 for KEGG and 5.6e-7 for Reactome) were retrieved (Figure 3). Notably, these two pathways were considered in the initial review of the AOP 441, but not included due to lack of scientific evidence. The global approach used here can thus provide additional mechanistic knowledge to the existing AOP. Many cyclin-dependent kinase genes (e.g., CDK2; CDK6; CDC6) are involved in these pathways (Figure 4). Cyclin-dependent kinases are responsible for TP53-mediated cell cycle arrest via P21 (CDKN1A) (Liu et al, 2020), but also appear to be involved in non-TP53 dependent pathways. Indeed, it was observed a downregulation of Cdk2 and Cdc6 after irradiation of TP53 null mutant mouse embryos. The downregulation of these Cyclin-dependent kinases can be explained by an upregulation of some members of the E2F transcription factors family, therefore suggesting an alternative pathway of TP53 leading to cell cycle arrest (Verheyde et al, 2006; Verheyde and Benotmane, 2007). Cell cycle arrest leads then to a decrease in cell proliferation, impacting the proper development of the brain and therefore representing an additional risk factor for microcephaly (Barkovich et al, 2005; Francis et al, 2006; Passemard et al, 2013). From these data, it is therefore possible to add the two additional KE 1505 “Cell cycle, Disrupted” and KE 1821“Decrease, cell proliferation” the existing AOPs for microcephaly (Figure 5).

Moreover, a wide range of genes present in the network are found differentially expressed in irradiated rodent animal models (Verheyde et al, 2006; Quintens et al, 2015; Mfossa et al, 2020). For example, the p53 activation following DNA damage triggered by IR leads to the induction of genes associated with differentiation and development processes, responsible for premature differentiation of neuron progenitors (Quintens et al, 2015). The key gene ASPM, known to be responsible for primary microcephaly when the protein is disrupted (Bond et al, 2003; Létard et al, 2018) was also found by our approach and linked to IR. On different human and murine cell lines as well as on fetal mouse brain, a study has shown that IR lead to a decrease in ASPM mRNA level associated with a reduction of ASPM protein (Fujimori et al, 2008). Moreover, it was observed that this downregulation of ASPM by IR was accompanied by a reduction of BRCA1, also present in our network. ASPM has many roles explaining its involvement in the process of microcephaly. This protein is involved in brain development by maintaining neural progenitor division, proliferation and differentiation (Fujimori et al, 2014; Létard et al, 2018) and is also required for the proper functioning of DNA repair. A recent study showed that ASPM facilitated DSB repair by stabilizing BRCA1 and protecting it from degradation by the proteasome, and that inhibition of ASPM expression led to an increase in BRCA1 ubiquitination leading to a decrease in its protein levels (Xu et al, 2021). Moreover, downregulation of Aspm was also observed in embryonic murine p53 null, meaning that the alteration of its expression is not or not totally due to TP53 activation (Fujimori et al, 2008).

Thus, a new KE “Alteration of gene expression” could be proposed for our AOP, which would lead to cell cycle arrest (Verheyde et al, 2006; Mfossa et al, 2020), impaired proliferation and differentiation of neural stem/progenitors cells (Verheyde et al, 2006; Fujimori et al, 2014; Quintens et al, 2015; Létard et al, 2018; Mfossa et al, 2020), as well as inadequate DNA damage repair (Xu et al, 2021) (Figure 5). Indeed, defective DNA repair is an important risk factor in microcephaly process and can amplify all the KEs of the AOP (i.e., excessive activation of TP53; apoptosis; premature cell differentiation; cell cycle arrest etc.). For example, DNA damage repair defects due to PNPK mutations (Shen et al, 2010) or DNA ligase IV deficiency (Chrzanowska et al, 2012) found in our network, result in radiosensitivity and can lead to the development of microcephaly.

The information on the newly identified potential KEs could be completed by using the KERs already present on AOP-Wiki. Thus, a two-way relationship between “Increased DNA damage” and “Inadequate DNA repair” described through KERs 1910, 1911 and 2,608 could be added, supporting the notion of an excessive accumulation of DNA damage leading to an overwhelmed DNA repair process. Relationships between “Increased DNA damage” and “Altered Gene expression” through KER 1301 or between “Increased DNA damage” and “Cell cycle, Disrupted” through KER 2790 could also be added, suggesting in particular that the modification of the expression of some key genes is not totally due to TP53 activation.

## 4 Discussion

In recent years, there has been an exponential increase in scientific literature focusing on toxicological mechanisms (INERIS, 2021). As a result, there is a need to promote the development of rapid approaches for identifying and correlating this information. Therefore, the concept of AOPs provides a coherent framework for organizing toxicological data at different levels of biological organization, serving as a useful tool for bridging the scientific and regulatory communities, while also highlighting areas of uncertainty and current research needs (Ankley et al, 2010; INERIS, 2021). AOPs are globally endorsed by the Organisation for Economic Co-operation and Development (OECD), which plays a central role in promoting AOPs through the establishment of development guidelines (OECD, 2018), reports, and community platforms (e.g., AOP-Wiki). Additionally, the OECD validates and harmonizes AOPs to ensure their scientific quality and relevance, thereby enhancing their credibility, particularly within regulatory frameworks. Despite the existence of guidelines, ensuring the proper development of an AOP remains a time-consuming manual process, essential for establishing a comprehensive set of biological evidence. To accelerate this process, there is a need for automated approaches (Ramšak et al, 2022). This systems biology approach aligns with this requirement by enabling the collection and analysis of a large volume of data from diverse sources, supporting the development of AOPs and, consequently, New Approaches Methodologies (NAMs) in toxicology. The strength of this approach lies in its simplicity by using only 2 keywords (i.e., the name of the stressor and the adverse outcome of interest), thus allowing any scientist wishing to develop or enrich his AOP to apply it.

The presented case study on the AOP 441 illustrates this approach. The combined use of databases and AOP-helpFinder enabled the identification of genes and pathways that are causative for microcephaly and associated with IR exposure, highlighting that both are heavily linked to DNA damage and TP53. Indeed, starting only with the keywords “microcephaly” and “ionizing radiation,” we were able to identify a set of 101 genes associated with these terms. Among them, a large number of genes were linked to the response to radiation-induced DNA damage and subsequent activation of TP53, including ATM, ATR, RAD50, MRE11, BRCA1, and TP53 itself. The direct causal association of these genes with microcephaly remain however to be assessed more precisely. The classification by biological enrichment analysis of all these identified genes also demonstrated that the most significant pathways were those related to DNA damage, TP53 activation, and apoptosis, which aligned with the key events (KEs) identified by experts during the development of AOP 441. The direct implication of neuronal progenitors death by apoptosis is largely documented as one of the most important mode of action leading to microcephaly. However, it is important to note that the time window of exposure to IR is critical, and that different embryonic stages display different sensitivity to irradiation. Indeed, microcephaly is only observed after exposure throughout the neurogenesis period where neuron are produced from progenitors, from E11 to E17 in the mouse (weeks 8 and 25 of gestation in human). When developing AOP, care must thus be taken to describe appropriately the biological conditions (time of development, tissue, condition of exposure, potential difference between sex, etc.) for which the adverse outcomes are expected. Such applicability domain definition is particularly important in the case of system biology approaches where such information are usually not provided.

In addition, the approach allowed the identification of 4 potential new KEs: altered gene expression, cell cycle disrupted, inadequate DNA repair and altered proliferation. The analysis of the gene-pathway network combined with the literature provided by AOP-help Finder allowed to propose new KERs enriching the AOP 441, transforming it into a more complex AOP network (AON). In total, 8 new KERs could be proposed, to which we can add 4 KERs already described on AOP-Wiki, bringing the total to 12 new links. The method presented here, can thus be particularly useful to produce rapidly novel preliminary AOP and novel potential KE and KER. Care must be taken to validate these results through expert review of the literature of the weight of evidence and describing appropriate biological applicability domain (as described above). Such assessment is not yet subjected to automation but recent advances on artificial intelligence and the usage of “Foundation” model might ease this process in the future (Moor et al, 2023).

Although this systems biology approach is very promising by quickly and easily identifying potential KEs, it does have some limitations that users must keep in mind if they wish to use it for the development of their AOP. First, this strategy simply extracts data from databases without assessing their quality. To counter this limitation and reduce the risk of false positives, we chose to apply a filter by keeping only the genes containing more than 2 bibliographic references associated with our adverse outcome, i.e., microcephaly. Furthermore, the type of study linking stressor, genes, and AO is not taken into account. For example, here, a gene that has shown altered expression at the RNA level following exposure to IR has the same weight as a gene found in genetic or epidemiological studies. Moreover, it is important to note that the genes identified by our strategy are not necessarily genes directly responsible for radio-induced microcephaly. For instance, a broad range of identified genes are involved in the process of radiation-induced microcephaly, such as genes involved in DNA damage response, providing insights into the progression towards microcephaly but not representing a direct causal link. Additionally, some of the identified genes correspond to genes implicated in the etiology of microcephaly when mutated, such as MCPH1. Thus, these different points help to explain why some less interesting genes can be found in the network. It is also important to note that KE 1560 present in the AOP441, namely, “Activation of TP53 leads to Premature differentiation,” is not detected in the present analysis, presumably due to the low number of publication available to describe this process.

In conclusion, this strategy is positioned as a rapid approach to assist in the development and enrichment of AOPs, but is not designed for automatic development of AOPs and requires the expertise of scientists to prioritize and integrate the results and literature obtained. This approach is versatile and can be applied in different situations, which has been demonstrated in the case study. It can be used to identify preliminary KEs before starting the development of the AOP through the identification of pathways, to identify new KEs, but also to obtain relevant bibliographic support through the use of text mining to facilitate the development of the AOP.

## Data Availability

The original contributions presented in the study are included in the article/Supplementary Material, further inquiries can be directed to the corresponding author.
