# Supplementary material for: An integrative systems biology strategy to support the development of adverse outcome pathways (AOPs): a case study on radiation-induced microcephaly
Source: Front Cell Dev Biol. 2023 Jun 22;11:1197204. doi: 10.3389/fcell.2023.1197204 (PMC10323360; doi:10.3389/fcell.2023.1197204)
Supplement: Supplementary file 2 [file Table1.DOCX]

Supplementary Material

An integrative systems biology strategy to support the development of Adverse Outcome Pathways (AOPs): a case study on radiation-induced microcephaly.

Thomas Jaylet, Roel Quintens, Olivier Armant, Karine Audouze*

*** Correspondence:** Karine Audouze: [karine.audouze@u-paris.fr](mailto:karine.audouze@u-paris.fr)

**Figure S1.** Distribution of microcephaly-associated genes extracted from DisGeNET **(A)** and GeneCards **(B)** according to their number of microcephaly-associated bibliographic supports referenced on these databases.

**Figure S2:** Biological enrichment results for WikiPathways (09/2022 release) for the list of 101 genes common to microcephaly and ionizing radiation extracted by our computational strategy.

**Table S1:** List of 382 genes associated with microcephaly extracted from DisGeNET and GeneCards after filtering. The columns "Related PMIDs” (GeneCards, DisGeNET) provide information about the number of publications associating the gene with microcephaly. The columns "Related PMIDs" and "Score” (GeneCards, DisGeNET) provide information about the confidence level of the gene-microcephaly association. The column "AOP-helpFinder" indicates the genes for which an association with IRs has also been found (101 genes).

**Table S2:** Significant Pathways Relevant to Study from KEGG, WikiPathways, Reactome (January, 17, 2023)**.**


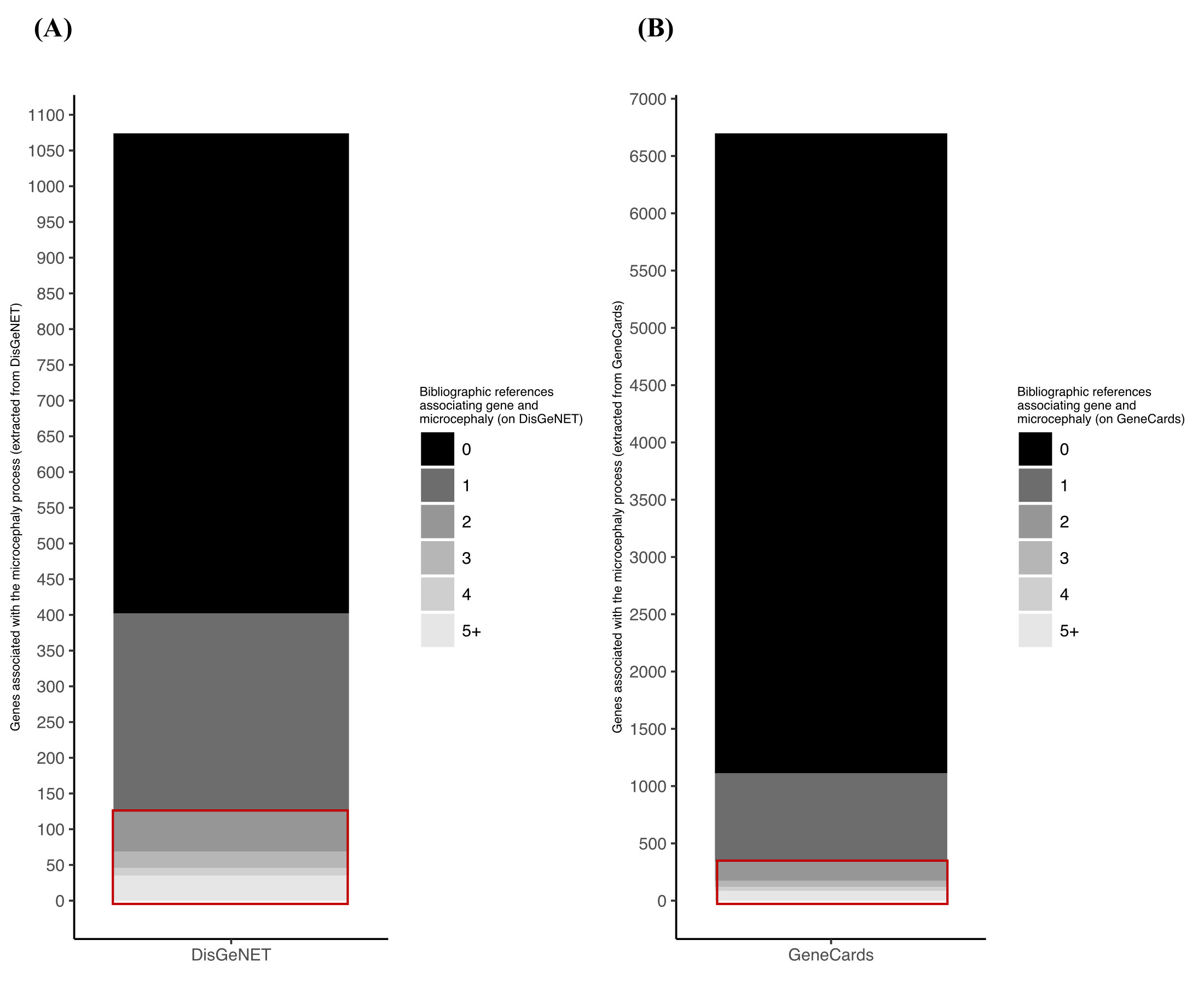
 **Figure S1.** Distribution of microcephaly-associated genes extracted from DisGeNET **(A)** and GeneCards **(B)** according to their number of microcephaly-associated bibliographic supports referenced on these databases. For the study, only genes containing more than 2 bibliographic supports associated with microcephaly were kept (boxed in red), representing a total of 117 out of 1064 genes for DisGeNET and 346 out of 6708 genes for GeneCards.

**
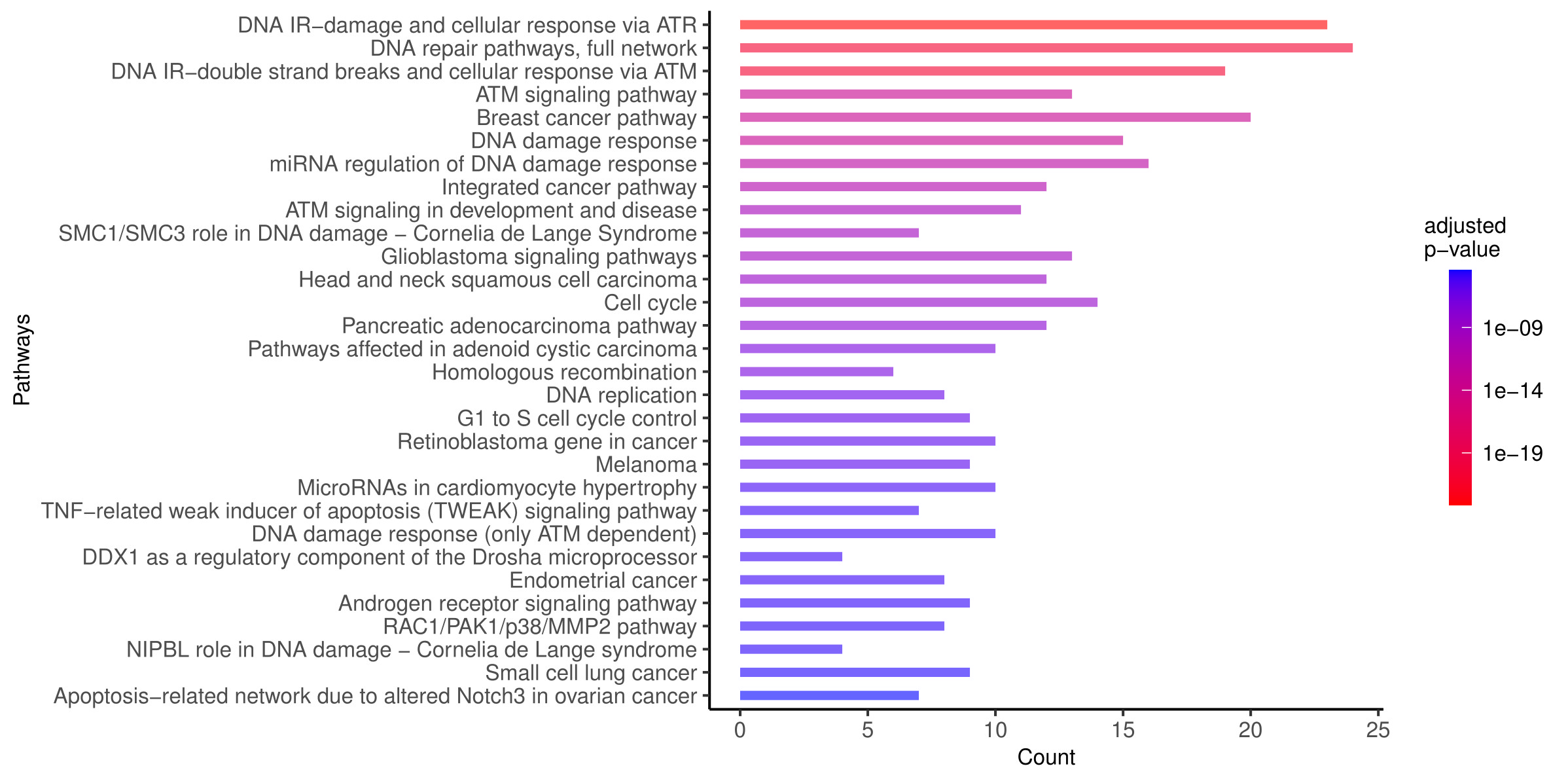
Figure S2:** Biological enrichment results for WikiPathways (09/2022 release) for the list of 101 genes common to microcephaly and ionizing radiation extracted by our computational strategy. Only the top 30 terms are shown.

List of 382 genes associated with microcephaly extracted from DisGeNET and GeneCards after filtering. The columns "Related PMIDs” (GeneCards, DisGeNET) provide information about the number of publications associating the gene with microcephaly. The columns "Related PMIDs" and "Score” (GeneCards, DisGeNET) provide information about the confidence level of the gene-microcephaly association. The column "AOP-helpFinder" indicates the genes for which an association with IRs has also been found (101 genes).

Separate excel file (TableS1.xlsx)


**Table S2:** Significant Pathways Relevant to Study from KEGG, WikiPathways, Reactome (January, 17, 2023)

| **Term** | **Adjusted p-value** | **Genes** |
| --- | --- | --- |
| **KEGG** | | |
| **4.2 Cell growth and death** | | |
| Cellular senescence | 5.48^e^-10 | AKT1,AKT3,ATM,ATR,CDK2,CDK6,CHEK1,GATA4,MRE11,NBN,NFKB1,  PIK3R1,PTEN, RAD50,RB1,TP53 |
| Cell cycle | 1.65e-9 | ATM,ATR,CDC6,CDK2,CDK6,CHEK1,MCM2,MCM7,ORC4,PCNA,PLK1,RB1,SMC3,TP53 |
| Apoptosis | 1.99e-7 | ACTB,AKT1,AKT3,ATM,EIF2AK3,ERN1,MAPK8,NFKB1,PARP1,PIK3R1,TNF,TP53 |
| p53 signaling pathway | 6.39e-6 | ATM,ATR,CDK2,CDK6,CHEK1,IGF1,PTEN,TP53 |
| Necroptosis | 0.044 | H2AX,MAPK8,PARP1,TLR3,TNF |
| **2.4 Replication and repair** | | |
| Homologous recombination | 6.49e-10 | ATM,BRCA1,BRCA2,BRIP1,MRE11,NBN,RAD50,RAD51,RAD51C,RBBP8 |
| Fanconi anemia pathway | 7.56e-8 | ATR,ATRIP,BRCA1,BRCA2,BRIP1,ERCC1,FANCD2,RAD51,RAD51C |
| Base excision repair | 0.001 | MBD4,PARP1,PCNA,XRCC1 |
| Non-homologous end-joining | 1.49^e^-6 | LIG4,MRE11,NHEJ1,RAD50,XRCC4 |
| DNA replication | 0.013 | MCM2,MCM7,PCNA |
| Nucleotide excision repair | 0.023 | ERCC1,ERCC5,PCNA |
| **6.7 Neurodegenerative disease** | | |
| Alzheimer disease | 0.002 | AKT1,AKT3,APOE,APP,CTNNB1,EIF2AK3,ERN1,MAPK8,MT-ATP6,NFKB1, PIK3R1,TNF |
| Pathways of neurodegeneration - multiple diseases | 0.043 | APP,CTNNB1,EIF2AK3,ERN1,MAPK8,MT-ATP6,NFKB1,RAC1,TNF,UBC |
| **Wikipathways** | | |
| **Related to DNA damage / repair** | | |
| DNA IR-damage and cellular response via ATR | 6.98e-24 | ATM,ATR,ATRIP,BRCA1,BRCA2,BRIP1,CDK2,CHEK1,FANCD2,H2AX,MCM2,MCPH1,MDC1,MRE11,NBN,PARP1,PCNA,PLK1,RAD50,RAD51,RBBP8,TP53,TP53BP1 |
| DNA IR-double strand breaks and cellular response via ATM | 1.39e-21 | ATM,ATR,BRCA1,BRCA2,CHEK1,FANCD2,H2AX,MCPH1,MDC1,MRE11,NBN,PARP1,PCNA,RAD50,RAD51,SMC3,TERF2,TP53,TP53BP1 |
| DNA repair pathways, full network | 1.39e-21 | ATM,ATR,BRCA1,BRCA2,BRIP1,CHEK1,ERCC1,ERCC5,FANCD2,H2AX,LIG4,MBD4,MRE11,NBN,NHEJ1,PARP1,PCNA,PNKP,RAD50,RAD51,RAD51C,  TERF2,XRCC1,XRCC4 |
| ATM signaling pathway | 3.97e-14 | ATM,BRCA1,CDK2,CHEK1,FANCD2,H2AX,MDC1,MRE11,NBN,RAD50,  RAD51,TP53,TP53BP1 |
| DNA damage response | 7.93e-14 | ATM,ATR,ATRIP,BRCA1,CDK2,CDK6,CHEK1,FANCD2,H2AX,  MRE11,NBN,RAD50,RAD51,RB1,TP53 |
| miRNA regulation of DNA damage response | 1.11e-12 | ATM,ATR,ATRIP,BRCA1,CDK2,CDK6,CHEK1,FANCD2,H2AX,  MCM7,MRE11,NBN,RAD50,RAD51,RB1,TP53 |
| ATM signaling in development and disease | 1.28e-10 | ATM,ATR,CHEK1,H2AX,MDC1,MRE11,NBN,NFKB1,RAD50,RBBP8,TP53BP1 |
| Homologous recombination | 1.039e-7 | ATM,BRCA2,MRE11,NBN,RAD50,RAD51 |
| DNA damage response (only ATM dependent) | 1.14e-5 | AKT1,AKT3,ATM,CTNNB1,MAPK8,NFKB1,PIK3R1,PTEN,RAC1,TP53 |
| Base excision repair | 3e-4 | MBD4,PARP1,PCNA,PNKP,XRCC1 |
| ATR signaling | 9.67e-4 | ATR,ATRIP,CHEK1 |
| SMC1/SMC3 role in DNA damage | 3.66e-10 | ATM,BRCA1,MDC1,MRE11,NBN,RAD50,SMC3 |
| Non-homologous end joining | 0.001 | LIG4,NHEJ1,XRCC4 |
| Nucleotide excision repair | 0.037 | ERCC1,ERCC5,PCNA |
| Oxidative damage response | 0.032 | NFKB1,PCNA,TNF |
| **Related to cell death / growth** | | |
| p53 transcriptional gene network | 9.86e-4 | AURKA,CCL2,CDK2,ERCC5,PCNA,PTEN,TNF |
| TP53 network | 0.049 | ATM,TP53 |
| Cell cycle | 3.28e-9 | ATM,ATR,CDC6,CDK2,CDK6,CHEK1,MCM2,MCM7,ORC4,PCNA,PLK1,RB1,  SMC3,TP53 |
| G1 to S cell cycle control | 1.23e-6 | ATM,CDK2,CDK6,MCM2,MCM7,ORC4,PCNA,RB1,TP53 |
| Apoptosis | 0.0006 | AKT1,IGF1,IGF1R,NFKB1,PIK3R1,TNF,TP53 |
| **Reactome** | | |
| **DNA repair** | | |
| DNA Double-Strand Break Repair | 8.01e-27 | ATM,ATR,ATRIP,BRCA1,BRCA2,BRIP1,CDK2,CHEK1,ERCC1,H2AX,KPNA2,LIG4,MAPK8,MDC1,MRE11,NBN,NHEJ1,PARP1,PCNA,PIAS4,RAD50,RAD51,RAD51C,RBBP8,TP53,TP53BP1,UBC,XRCC1,XRCC4 |
| DNA Repair  (TOP LEVEL PATHWAY) | 7.40e-26 | ACTB,ATM,ATR,ATRIP,BRCA1,BRCA2,BRIP1,CDK2,CHEK1,ERCC1,ERCC5,FANCD2,H2AX,KPNA2,LIG4,MAPK8,MBD4,MDC1,MRE11,NBN,NHEJ1,PARP1,PCNA,PIAS4,PNKP,RAD50,RAD51,RAD51C,RBBP8,TERF2,TP53,TP53BP1,UBC,XRCC1,XRCC4 |
| Homology Directed Repair | 1.14e-20 | ATM,ATR,ATRIP,BRCA1,BRCA2,BRIP1,CDK2,CHEK1,ERCC1,H2AX,MDC1,MRE11,NBN,PARP1,PCNA,PIAS4,RAD50,RAD51,RAD51C,RBBP8,TP53BP1,UBC,XRCC1 |
| HDR through Homologous Recombination (HRR) or Single Strand Annealing (SSA) | 1.85e-18 | ATM,ATR,ATRIP,BRCA1,BRCA2,BRIP1,CDK2,CHEK1,ERCC1,H2AX,MDC1,MRE11,NBN,PCNA,PIAS4,RAD50,RAD51,RAD51C,RBBP8,TP53BP1,UBC |
| HDR through Homologous Recombination (HRR) | 2.62e-15 | ATM,ATR,ATRIP,BRCA1,BRCA2,BRIP1,CHEK1,MRE11,NBN,PCNA,RAD50,RAD51,RAD51C,RBBP8,UBC |
| Processing of DNA double-strand break ends | 3.60e-14 | ATM,ATR,ATRIP,BRCA1,BRIP1,CDK2,CHEK1,H2AX,MDC1,MRE11,NBN,PIAS4,RAD50,RBBP8,TP53BP1,UBC |
| Defective HDR […] | 3.12e-13 | ATM,BRCA1,BRCA2,BRIP1,MRE11,NBN,RAD50,RAD51,RAD51C,RBBP8 |
| Diseases of DNA Double-Strand Break Repair | 3.12e-13 | ATM,BRCA1,BRCA2,BRIP1,MRE11,NBN,RAD50,RAD51,RAD51C,RBBP8 |
| DNA Double Strand Break Response | 8.59e-12 | ATM,BRCA1,H2AX,KPNA2,MAPK8,MDC1,MRE11,NBN,PIAS4,RAD50,TP53,TP53BP1,UBC |
| Nonhomologous End-Joining (NHEJ) | 3.93e-11 | ATM,BRCA1,H2AX,LIG4,MDC1,MRE11,NBN,NHEJ1,PIAS4,RAD50,TP53BP1,XRCC4 |
| Recruitment and ATM-mediated phosphorylation of repair and signaling proteins at DNA double strand breaks | 1.43e-10 | ATM,BRCA1,H2AX,MAPK8,MDC1,MRE11,NBN,PIAS4,RAD50,TP53,TP53BP1,UBC |
| HDR through MMEJ (alt-NHEJ) | 3.29e-9 | MRE11,NBN,PARP1,RAD50,RBBP8,XRCC1 |
| Base Excision Repair | 0.0002 | H2AX,MBD4,PARP1,PCNA,PNKP,TERF2,XRCC1 |
| Fanconi Anemia Pathway | 0.0004 | ATR,ATRIP,ERCC1,FANCD2,UBC |
| Nucleotide Excision Repair | 0.0009 | ACTB,ERCC1,ERCC5,PARP1,PCNA,UBC,XRCC1 |
| **Cell cycle** | | |
| Cell cycle (TOP LEVEL PATHWAY) | 1.43e-19 | AKT1,AKT3,ATM,ATR,ATRIP,ATRX,AURKA,BRCA1,BRCA2,BRIP1,CDC6,CDK2,CDK6,CDT1,CHEK1,ESCO2,H2AX,MCM2,MCM7,MCPH1,MDC1,MRE11,NBN,ORC4,PCNA,PIAS4,PLK1,RAD50,RAD51,RAD51C,RB1,RBBP8,SMC3,TERF2,TERT,TP53,TP53BP1,UBC,VRK1 |
| G2/M Checkpoints | 2.76e-16 | ATM,ATR,ATRIP,BRCA1,BRIP1,CDC6,CDK2,CHEK1,H2AX,MCM2,MCM7,MDC1,MRE11,NBN,ORC4,PIAS4,RAD50,RBBP8,TP53,TP53BP1,UBC |
| G2/M DNA damage checkpoint | 3.41e-13 | ATM,ATR,ATRIP,BRCA1,BRIP1,CHEK1,H2AX,MDC1,MRE11,NBN,PIAS4,RAD50,RBBP8,TP53,TP53BP1 |
| Cell Cycle Checkpoints | 6.27e-13 | ATM,ATR,ATRIP,BRCA1,BRIP1,CDC6,CDK2,CHEK1,H2AX,MCM2,MCM7,MDC1,MRE11,NBN,ORC4,PIAS4,PLK1,RAD50,RBBP8,TP53,TP53BP1,UBC |
| Activation of ATR in response to replication stress | 3.17e-8 | ATR,ATRIP,CDC6,CDK2,CHEK1,MCM2,MCM7,ORC4 |
| S Phase | 5.95e-8 | AKT1,AKT3,CDC6,CDK2,CDT1,ESCO2,MCM2,MCM7,ORC4,PCNA,RB1,SMC3,UBC |
| Mitotic G1 phase and G1/S transition | 2.17e-7 | AKT1,AKT3,CDC6,CDK2,CDK6,CDT1,MCM2,MCM7,ORC4,PCNA,RB1,UBC |
| G1/S Transition | 6.18e-7 | AKT1,AKT3,CDC6,CDK2,CDT1,MCM2,MCM7,ORC4,PCNA,RB1,UBC |
| Cell Cycle, Mitotic | 0.000002 | AKT1,AKT3,AURKA,CDC6,CDK2,CDK6,CDT1,ESCO2,H2AX,MCM2,MCM7,MCPH1,ORC4,PCNA,PLK1,RB1,SMC3,TP53,UBC,VRK1 |
| G1/S DNA Damage Checkpoints | 0.0045 | ATM,CDK2,CHEK1,TP53,UBC |
| G1 Phase | 0.0076 | CDK2,CDK6,RB1,UBC |
| Cyclin D associated events in G1 | 0.0076 | CDK2,CDK6,RB1,UBC |
| Cyclin E associated events during G1/S transition | 0.0085 | AKT1,AKT3,CDK2,RB1,UBC |
| Cyclin A:Cdk2-associated events at S phase entry | 0.0093 | AKT1,AKT3,CDK2,RB1,UBC |
| Regulation of mitotic cell cycle | 0.011 | AURKA,CDK2,PLK1,RB1,UBC |
| Chromosome Maintenance | 0.013 | ATRX,CDK2,H2AX,PCNA,TERF2,TERT |
| p53-Dependent G1 DNA Damage Response | 0.022 | ATM,CDK2,TP53,UBC |
| p53-Dependent G1/S DNA damage checkpoint | 0.022 | ATM,CDK2,TP53,UBC |
| **Gene expression (Transcription)** | | |
| Regulation of TP53 Activity | 3.24e-12 | AKT1,AKT3,ATM,ATR,ATRIP,AURKA,BRCA1,BRIP1,CDK2,CHEK1,KAT6A,MRE11,NBN,RAD50,RBBP8,TP53,UBC |
| Regulation of TP53 Activity through Phosphorylation | 3.83e-12 | ATM,ATR,ATRIP,AURKA,BRCA1,BRIP1,CDK2,CHEK1,MRE11,NBN,RAD50,RBBP8,TP53,UBC |
| Transcriptional Regulation by TP53 | 2.56e-10 | AKT1,AKT3,ATM,ATR,ATRIP,AURKA,BRCA1,BRIP1,CDK2,CHEK1,FANCD2,KAT6A,MDC1,MRE11,NBN,PCNA,PTEN,RAD50,RBBP8,TP53,UBC |
| Gene expression (Transcription) (TOP LEVEL PATHWAY) | 1.68e-8 | ACTB,AKT1,AKT3,APOE,ARID1B,ATM,ATR,ATRIP,AURKA,BRCA1,BRIP1,CDK2,CDK6,CGA,CHEK1,COL1A1,CTNNB1,FANCD2,GATA4,H2AX,KAT6A,MDC1,MRE11,NBN,NFKB1,OCLN,PARP1,PCNA,PTEN,RAD50,RAD51,RB1,RBBP8,SMARCA2,TGIF1,TP53,UBC,USP9X |
| Regulation of TP53 Degradation | 0.000017 | AKT1,AKT3,ATM,CDK2,TP53,UBC |
| Regulation of TP53 Expression and Degradation | 0.000020 | AKT1,AKT3,ATM,CDK2,TP53,UBC |
| TP53 Regulates Transcription of DNA Repair Genes | 0.000026 | ATM,ATR,BRCA1,CHEK1,FANCD2,MDC1,TP53 |
| Regulation of TP53 Activity through Acetylation | 0.002049 | AKT1,AKT3,KAT6A,TP53 |
| Transcriptional Regulation by E2F6 | 0.003160 | BRCA1,CHEK1,RAD51,RBBP8 |
| TP53 Regulates Transcription of Genes Involved in G2 Cell Cycle Arrest | 0.0058 | AURKA,PCNA,TP53 |
| Regulation of TP53 Activity through Methylation | 0.0065 | ATM,TP53,UBC |
| TP53 Regulates Transcription of Cell Cycle Genes | 0.0094 | AURKA,CDK2,PCNA,TP53 |
| TP53 Regulates Transcription of Caspase Activators and Caspases | 0.030 | ATM,TP53 |
| **Disease** | | |
| Diseases of DNA repair | 8.59e-12 | ATM,BRCA1,BRCA2,BRIP1,MRE11,NBN,RAD50,RAD51,RAD51C,RBBP8 |
| Diseases of mitotic cell cycle | 0.004498 | ATRX,CDK2,CDK6,RB1 |
| **Cellular responses to stimuli** | | |
| Cellular Senescence | 5.64e-7 | ATM,CDK2,CDK6,H2AX,MAPK8,MRE11,NBN,NFKB1,RAD50,RB1,TERF2,TP53,UBC |
| DNA Damage/Telomere Stress Induced Senescence | 0.000001 | ATM,CDK2,H2AX,MRE11,NBN,RAD50,RB1,TERF2,TP53 |
| Cellular responses to stimuli (TOP LEVEL PATHWAY) | 0.0057 | ATM,ATR,CCL2,CDK2,CDK6,EIF2AK3,ERN1,H2AX,MAPK8,MRE11,NBN,NFKB1,RAD50,RB1,TERF2,TP53,UBC |
| Cellular responses to stress | 0.0049 | ATM,ATR,CCL2,CDK2,CDK6,EIF2AK3,ERN1,H2AX,MAPK8,MRE11,NBN,NFKB1,RAD50,RB1,TERF2,TP53,UBC |
| **Programmed cell death** | | |
| Apoptosis | 0.000085 | AKT1,AKT3,BCAP31,CDH1,CTNNB1,MAPK8,OCLN,PAK2,TP53,UBC |
| Programmed cell death (TOP LEVEL PATHWAY) | 0.000292 | AKT1,AKT3,BCAP31,CDH1,CTNNB1,MAPK8,OCLN,PAK2,TP53,UBC |
| Apoptotic execution phase | 0.001395 | BCAP31,CDH1,CTNNB1,OCLN,PAK2 |
| Apoptotic cleavage of cell adhesion  proteins | 0.001622 | CDH1,CTNNB1,OCLN |
| Apoptotic cleavage of cellular proteins | 0.004197 | BCAP31,CDH1,CTNNB1,OCLN |
| **Signal Transduction** | | |
| Signal Transduction (TOP LEVEL PATHWAY) | 0.005697 | ACTB,AKT1,AKT3,APOE,APP,AXL,BCAP31,CCL2,CDH1,CDK2,CGA,CHEK1,COL1A1,CTNNB1,DLD,EDNRB,GDNF,H2AX,IGF1,IGF1R,KPNA2,MAPK8,MYCN,NFKB1,PAK2,PARP1,PIK3R1,PLK1,PTEN,RAC1,SHH,SMC3,TERT,TGIF1,TNF,TP53,UBC,USP9X |
